# Hormone-Driven Growth Signaling as a Therapeutic Target in Acute Myeloid Leukemia: Implications for Drug-Resistant Disease

**DOI:** 10.3390/jpm16060331

**Published:** 2026-06-20

**Authors:** Joel Costoya, Joaquin J. Jimenez

**Affiliations:** 1 Department of Biochemistry and Molecular Biology, Miller School of Medicine, University of Miami, Miami, FL 33136, USA; 2 Dr. Phillip Frost Department of Dermatology and Cutaneous Surgery, Miller School of Medicine, University of Miami, Miami, FL 33136, USA

**Keywords:** AML, MIA-602, GHRH antagonism, drug-resistance, *FLT3*

## Abstract

Growth hormone-releasing hormone (GHRH) antagonists have displayed anti-neoplastic activity against a multitude of cancers in vitro, as well as in vivo, via xenografted tumors in nude mice. Following a successful demonstration of GHRH antagonists treating non-Hodgkin’s lymphoma and the discovery of *GHRH* mRNA and peptide products in immune cells, GHRH antagonism was explored in acute myeloid leukemia (AML), a disease characterized by a malignant expansion of immature myeloid progenitors, and poor 5-year survival. Targeted therapies have yielded breakthroughs in treatment response and overall survival, such as all-trans retinoic acid/arsenic trioxide (ATRA/ATO) for acute promyelocytic leukemia (APL), or FLT3 inhibitors, IDH inhibitors, and menin inhibitors for AML harboring actionable genetic lesions. However, therapeutic resistance remains a major barrier to durable remission. GHRH receptor (GHRH-R) has been reported in several experimental models of AML, including drug-resistant sublines. Significant time- and dose-dependent reduction in leukemic growth was observed in vitro and in vivo following MIA-602 treatment. FLT3 inhibitor resistance has been associated with activation of PI3K/AKT, ERK/MAPK, inflammatory, stromal, and apoptotic escape pathways. The documented effects of GHRH-R antagonism raise the possibility that it could influence signaling networks relevant to therapeutic resistance in AML. This hypothesis remains speculative; to date no studies have stratified AML by *FLT3* status in the context of GHRH-R expression or GHRH antagonism, and there is currently no evidence that MIA-602 directly alters FLT3 receptor signaling or inhibitor sensitivity.

## 1. Introduction

AML is a highly heterogenous hematological malignancy characterized by a series of aberrations to genetic programs responsible for the proliferation, maturation, and survival of myeloid progenitor cells during the process of hematopoiesis [1]. Clonal expansion of immature myelocytic cells occurs in the bone marrow, crowding out other blood cell populations and eventually spreading into the peripheral blood and throughout the body. Concurrently, bone marrow dysfunction develops, often resulting in patients presenting emergently with various associated cytopenias and transfusion dependency [1,2]. A primary diagnostic criterion for AML is a myeloblast count of 20% or higher found in the peripheral blood or bone marrow, although there are several cases of specific cytogenetic abnormalities such as AML with *RUNX1::RUNX1T1* fusions, *CBFB::MYH11* fusions, *PML::RARA* fusions, *RBM15::MRTFA* fusions, *DEK::NUP214* fusions, and AML with *KMT2A* rearrangements, *MECOM* rearrangements, *NUP98* rearrangements, or AML with *NPM1* mutations, that serve as exceptions to the typical blast percentage rule [3,4,5]. The diagnostic work-up for a suspected AML incorporates morphological evaluation of the blasts, assisted by flow cytometric immunophenotyping to help confirm the identity of specific AML subtypes and establish baseline measurements for minimal residual disease tracking. Furthermore, pertinent diagnostic and prognostic information ought to be extracted from genetic studies like karyotyping, fluorescence in situ hybridization and next-generation sequencing, as genomic annotation usually will be most indicative of treatment outcomes and will help guide selecting optimal therapeutic regimens [1,2,6,7].

The prognosis for AML is usually poor, and in addition to its classification, is largely influenced by patient characteristics such as any existing co-morbidities and age. Despite continuous improvement in AML therapies there still remain significant hurdles to achieving complete remission post-therapy, as a large fraction of patients experience relapse [1]. According to the SEER U.S. database, amongst adult populations, AML is the most prevalent leukemia, exhibiting a five-year survival rate of 31.7% [8]. Induction therapy for newly diagnosed AML aims to achieve complete remission through intense chemotherapy regimens, most commonly using the “7 + 3” approach, consisting of administering daunorubicin for 3 days and cytarabine for 7 days. For patients deemed unable to withstand more intense chemotherapy regimens, there are less-intense chemotherapeutic alternatives which are attempted in order to induce and maintain remission. The standard-of-care chemotherapeutic regimen combines a hypomethylating agent and venetoclax and is administered continuously with dose adjustments to minimize toxicity and allow for greater marrow recovery between cycles [2,9,10,11,12]. If during the initial work-up, the patient is discovered to have a treatable molecularly defined subset of AML, such as mutated fms-like tyrosine kinase 3 (*FLT3*), mutated isocitrate dehydrogenase 1/2 (*IDH1/2*), core-binding factor (*CBF*) AML, and Nucleophosmin 1 (*NPM1*)/Lysine Methyltransferase 2A (*KMT2A*) rearranged AML, then the front-line treatment strategies may be modified to incorporate targeted agents that capitalize on the vulnerabilities of the underlying biology. Organized with respect to the aforementioned genetic lesions, one could expect FLT3 inhibitors, IDH 1/2 inhibitors, gemtuzumab ozogamicin, or menin inhibitors, to be deployed alongside standard chemotherapy regimens. Currently, the only FDA-approved front-line therapies for patients eligible for intensive chemotherapy are FLT3 inhibitors and gemtuzumab ozogamicin, an antibody-drug conjugate targeting CD33. In the case of patients who are ineligible for intensive chemotherapy, their therapeutic regimen could include an FLT3 inhibitor in the case of *FLT3*-mutated AML. If the patient has an *IDH1* mutation, ivosidenib is now incorporated into their treatment plan as a monotherapy or in combination with a hypomethylating agent and/or venetoclax. To our knowledge, no FDA approvals have been given regarding IDH2 inhibitors as a front-line therapeutic; however, enasidenib is available in relapsed/or refractory (R/R) cases. At present, menin inhibitors are only FDA-approved to treat *KMT2A*-rearranged or *NPM1*-mutated AML in the relapsed/refractory setting; however, studies are underway exploring these targeted therapies in combination with front-line treatment regimens in patients eligible for intense chemotherapy and in the non-eligible setting where they are instead combined with hypomethylating agents and venetoclax. Patients suspected of having acute promyelocytic leukemia (APL) are to be started immediately on all-*trans* retinoic acid (ATRA) and then combined with arsenic trioxide (ATO) once it is confirmed they possess a *PML::RARA* fusion gene. These patients would forgo cytotoxic therapy unless considered “high risk” and in need of cytoreductive measures. Following remission, treatment is continued with consolidation chemotherapy and, in some cases, allogeneic hematopoietic stem cell transplantation [13]. Within the evolving framework of AML management, addressing therapeutic resistance remains a critical factor for achieving durable remissions and improving patient outcomes.

Advancements in diagnostic modalities have not only guided therapeutic strategies and the development of targeted drug therapies but also facilitated a shift towards precision medicine. A well-known example is found with the use of ATRA and ATO to treat APL, a rare variant of AML [14]. First described in 1957 by Hillestad as having a rapidly fatal course and considered the ‘most malignant form of acute leukemia’ [15] APL now has the most favorable prognosis of all AML. As with other subtypes of AML, acquired resistance remains a significant barrier to long-term disease-free survival, driven by the emergence of leukemic clones with the capacity to circumvent or tolerate these differentiating agents. Pre-clinical evidence of MIA-602 has shown significant anti-neoplastic activity against drug-resistant analogs of both AML and APL whilst mitigating peripheral toxicities. Interestingly, when administered in combination with currently used therapeutic agents, a potent synergistic effect was noted [16]. Here, we review and organize the pre-clinical evidence surrounding the GHRH-R antagonist “MIA-602”, as an investigational approach in AML and APL models, with emphasis on drug-resistant disease biology. Rather than imply established clinical efficacy, further mechanistic study of GHRH-R antagonism’s reported anti-neoplastic effects in AML may generate testable hypotheses for future AML studies due to overlapping biological signaling, implicated in resistance to targeted therapies like that of FLT3 inhibitors.

## 2. GHRH and Cancer: Impetus for Targeted Therapy in Acute Myeloid Leukemia

GHRH was first fully isolated and characterized in 1982, with early evidence of its existence within animal hypothalamic extracts spanning back more than half a century [17,18]. Interestingly, studying secretions of pancreatic islet tumors associated with acromegaly led to the elucidation of GHRH’s 40- and 44-amino acid isoforms, allowing for its full structural analysis and bioactivity probing [19]. Subsequent investigation across various animal species, as well as humans, confirmed the hypothalamus to be the primary site of its biogenesis. For several decades GHRH was viewed rather restrictively as a regulator of the pituitary GH/hepatic insulin-like growth factor 1 (IGF-1) axis, despite owing its discovery to ectopic tumoral productions. The recent literature points to GHRH being a much more pleiotropic peptide, exerting growth factor-like effects in a vast number of peripheral tissues, calling into question its identity being perhaps more than just that of a neuroendocrine hormone responsible for the release of GH from the pituitary [20,21]. Indeed, many extrahypothalamic tissues are known to synthesize GHRH, capable of acting in an autocrine/paracrine manner, logically implying the presence of GHRH-R activity. In corroboration, both GHRH-R and its splice variants (SVs) have been documented throughout various tissue types. The same phenomenon has been shown to occur in pathological states of varying cancers [21,22].

GHRH-R is a class B G protein-coupled receptor (GPCR) pertaining to the secretin-like sub-family, including but not limited to glucagon, glucagon-like peptides, pituitary adenylate cyclase-activating polypeptides, secretin, and vasoactive intestinal peptides. GHRH-R is composed of seven transmembrane helices with three intracellular and three extracellular loops, most highly expressed in the somatotrophs of the anterior pituitary gland (pGHRH-R), in line with its physiological role as a neuroendocrine hormone receptor [23]. To date, four SVs of GHRH-R have been documented in a multitude of both normal and neoplastic tissues, with SV1 acting as the main variant responsible for GHRH’s bioactivity and prime oncological target [24,25]. SV1 while largely similar to full-length GHRH-R lacks a portion of its extracellular domain as the result of an intron splicing event in where a fragment of intron 3 replaces the first three exons, and as such the first 89 amino acids are exchanged for a distinct 25-long amino acid sequence. The changes occurring near the N-termini of these receptors account for the difference in signaling observed between SV1 and its full-length counterpart [26,27]. Namely, GHRH-R is more Gs/cyclic adenosine monophosphate (cAMP)-dominant and responsible for inducing GH synthesis and release upon ligand stimulation, while SV1 displays more bias towards ß-arrestin signaling, consequentially exerting its mitogenic effects primarily through this pathway [23,27]. While still canonically capable of activating the cAMP/protein kinase A (PKA) cascade, SV1’s ß-arrestin bias promotes oncogenesis via increases in MAPK, ERK1/2, and PKA activity subsequent to arrestin recruitment [23,27,28]. In addition, it should be mentioned SV1 has been demonstrated to promote growth both via a ligand-dependent and ligand-independent manner [29].

Canonically, GHRH binding to GHRH-R activates the GPCR leading to increased cAMP, which activates PKA causing sodium channel-induced membrane depolarization followed by a calcium influx and subsequent GH secretion [23,30]. Concurrently, activated PKA signals CREB, a cAMP response element transcription factor, accompanied by cofactors such as p300 and CREB-binding protein to bind to cAMP response elements along the GH promoter region stimulating its transcription. GHRH-R can also activate phospholipase C yielding diacylglycerol and inositol triphosphate, of which the latter can increase intracellular calcium and lead to GH secretion similar to that induced by cAMP/PKA signaling [23]. Another notable pathway affected by GHRH-R activation is the protein kinase C-dependent stimulation of MAPK/ERK which ultimately leads to cell growth and proliferation [23].

Originally, the mechanism of action behind GHRH’s growth factor-like behavior was thought to arise from endocrine stimulation of IGF-I/II production through the pit-GH/hepatic-IGF axis, eliciting IGF-I/II’s potent mitogenic effects of enhanced cell growth, proliferation, and cellular survival. Although in vitro experimentation, naturally devoid of the pit-GH/hepatic-IGF axis, has shown GHRH to promote biogenesis of IGF-I/II in an autocrine/paracrine manner [31]. Additionally, GHRH-R activation has been shown to significantly stimulate growth independent of IGF signaling, relying on activation of MEK/ERK ½ and PI3K/AKT through alternative pathways [32]. The current rationale for how GHRH directly exerts its mitogenic effects in the absence of its endocrine signaling has been narrowed down to two main mechanisms. The first would be local production of GHRH by peripheral tissues which upon release from their originating cell can either self-induce, or stimulate nearby cells to synthesize and excrete GH, which then would, in an autocrine/paracrine manner, promote IGF-I/II production and form a potent GHRH- or IGF-stimulatory loop. This autocrine/paracrine stimulatory loop is of great importance given these hormones’ independent abilities to promote cell growth, survival, differentiation, and the physiological role they play in tissue-specific processes. Secondly, across various tissue types direct GHRH-R stimulation can modulate pathways including but not limited to those that control inflammation, motility, cell cycle dynamics, and angiogenesis, which can also serve as enhancers or promotors of mitogenic signaling [21,23,33]. Apart from GHRH’s canonical role in physiology, these signaling pathways can be used aberrantly by cancer cells for the purposes of promoting tumorigenesis and malignancy as shown across various cancer types experimentally. Therefore, direct GHRH autocrine/paracrine hormonal action has become increasingly relevant in the field of cancer, as exemplified by GHRH antagonists, synthetic analogs of this peptide hormone with GHRH-R antagonistic activity [33].

Engineered in hopes of creating novel anti-neoplastic agents, GHRH antagonists have since proved to be remarkably successful in the pre-clinical setting against a plethora of cancers, such as lung [34,35], breast [36,37,38], prostate [39,40,41], pancreas [42], retina [43], ovary [44], colon [45], brain [37,46], kidney [47], stomach [48], bone [49], endometrium [50], and lymphoma [51]. The mechanism by which GHRH antagonists function as anti-neoplastic agents is three-fold and is reliant upon competitive binding of the GHRH-R and its SVs, mainly SV1, such that the synthetic GHRH analog functionally antagonizes the receptors preventing their activation and allowing for subsequent inhibition of their downstream signaling [33]. Indirectly, GHRH antagonists can cause a decrease in available systemic GH and IGF-I/II, well-known tumorigenic agents, and thus prevent further oncogenic signaling that would be permissive for the growth and spread of malignancy throughout the body. Of particular relevance, the degree of endocrine suppression of the pituitary GH/hepatic IGF axis varies greatly depending on which GHRH antagonist is used, such that of the newest GHRH antagonists, some have displayed significantly weaker endocrine suppressive activity, whilst others have demonstrated far greater suppression. Currently no significant adverse health effects have been noted from the use of this anti-hormone targeted therapy in pre-clinical animal modeling, yet long-term safety data, as well as feasibility of use in a clinical setting remains unknown given the fact that GHRH antagonism has yet to be realized in human clinical trials [21,33]. On the other hand, GHRH antagonists can exhibit their anti-neoplastic activity on cancerous tissue by directly binding to GHRH receptors, forming a receptor blockade, and preventing tumor-derived GHRH from binding to its cognate receptor, diminishing the mitogenic effects of GHRH-R stimulation as well as subsequent tumoral IGF production that would have occurred as a result of GHRH-R activation, effectively cutting out two stimulatory loops in one. Another outcome of direct tumoral GHRH-R antagonism which adds to their efficacy as anti-neoplastic agents involves the inhibition of alternative downstream signaling, caused, unlike GH/IGF production, by tumor-derived GHRH/GHRH-R which otherwise would result in tumoral progression [23,33]. This is explained in more detail in the following text.

GHRH antagonists, in addition to inhibiting the MEK/ERK ½ and PI3K/AKT pathways [31], have also been shown to regulate telomeric activity [46], attenuate epithelial–mesenchymal transition and cellular motility pathways [37], modulate inflammatory cytokines [36], interfere with angiogenic factors [35,40], downregulate PAK-1/STAT3 [48], hinder release of cAMP [39] and impede cell cycle progression pathways [34], increase pro-apoptotic mechanisms [43] and decrease cell proliferation and tumorigenesis [16], resulting in their success as anti-cancer agents. Progressive optimization of these peptide hormone analogs has since produced increasingly potent oncolytic agents with decreased cytotoxicity in off-target tissues [34,50,52,53]. The use of GHRH antagonists against various cancer models is briefly summarized in Table 1.

Bioactive GHRH peptides and the expression of *GHRH* mRNA was confirmed in monocytes, B and T cell lymphocytes in primary samples of peripheral blood mononuclear cells [54]. It was also observed that the myeloid cell lines U937 and HL-60 displayed expression of *GHRH* mRNA, with U937 expressing a 2-fold increase in *GHRH* mRNA in comparison to primary monocytes [55]. Another study showed significant efficacy in treating experimental non-Hodgkin’s lymphoma both in vitro and in vivo using GHRH antagonists MZ-5-156 and MZ-J-7-138 [51]. Unlike MZ-5-156, despite having no inhibitory effect on hepatic IGF-I production or serum IGF-I, MZ-J-7-138 showed significantly stronger anti-tumoral effects likely owed to its higher binding affinity and direct GHRH-R interactions [51]. These findings spurred investigation into utilization of a newer GHRH antagonist MIA-602 as a possible therapeutic agent against AML.

MIA-602 represents one of the more recent iterations of the GHRH antagonists, specifically hailing from the Miami (MIA) series. Synthesis of these peptides occurs via solid-phase peptide methodology and *tert*-butoxycarbonyl(Boc)-chemistry. They are subsequently purified via elution from their synthesis resin using a trifluoroacetic acid (TFA) solvent system leading to formation of peptide salts. Finally analytical HPLC is utilized to achieve maximal purity (>95%), as previously described [50]. The “MIA” GHRH antagonists were developed by improving upon the peptide backbone of earlier “MZ-J” and “JMR” series antagonists, designed to enhance receptor binding affinity, bioavailability, tumor growth inhibition, and resistance to proteolytic degradation. These modifications came in the form of introducing N-terminal lipidation, further addition of non-natural amino acid substitutions, and conformational restrictions, whilst retaining some previous alterations to the peptide backbone such as the incorporation of D-Arg^2^, homoarginine^9^, histidine^11,20^, α-aminobutyric acid^15^, and norleucine^27^ due to observation that maintaining these changes preserved the core antagonistic activity, and conferred greater tumor inhibition, binding, and chemical stability in prior antagonists [50]. From a large pool of similar “MIA” antagonists, MIA-602 was chosen due to it showing one of the greatest receptor binding affinities, and anti-cancer activity both in vitro and in vivo. In contrast to earlier GHRH antagonists, MIA-602 exhibited substantially weaker endocrine GH suppression, suggesting the observed anti-neoplastic effects are working primarily through direct receptor antagonism and through systemic endocrine suppression to a much lesser degree [50].

The current newest class of GHRH antagonists belonging to the “AVR” series were developed following the introduction of several additional modifications to the MIA-602 scaffold and produced using solid-phase methodology and a 9-fluorenylmethoxycarbonyl (Fmoc) synthetic strategy as opposed to earlier Boc chemistry from the MIA series [56]. The result of adding fluorination to the N-terminal cap, adding a lipidic moiety to the C-terminus, fluorinating aromatic residues, and optimizing cationic residue placement yielded GHRH antagonists with ~2–4.5× higher binding affinities, and significantly greater anti-neoplastic activity in vitro and in vivo, of which AVR-352 and AVR-353 were the most notable when compared directly to MIA-602. Conversely, these AVR series antagonists exhibited greater GH endocrine suppression, successfully demonstrating this activity both in vivo and in vitro when compared with the prior generation MIA-602 [56]. These same AVR antagonists so far have been investigated for their anti-cancer activity against lung, gastric, pancreatic, colorectal, breast, glioblastoma, ovarian, and prostate cancer. Furthermore, these AVR series GHRH antagonists showed significantly greater anti-inflammatory activity than that of MIA-602 or methyl prednisolone in an in-mouse lung inflammatory model as measured by reduced expression of inflammation-associated cytokines [56]. However, due to the AVR antagonists’ limited primary data in comparison to the comparatively more robust literature documenting MIA-602’s pre-clinical efficacy, and the significantly greater endocrine GH suppression observed in these newer series peptides unlike their more unimodal predecessor, we will focus on the application of GHRH antagonism as it relates to AML using MIA-602 as the object of study.

**Table 1:** Use of GHRH antagonists against various cancer models.

Cancer Model and Notable GHRH Antagonist(s)	Pathway Affected	Observed Effects	Citation
AML/APL/Drug-Resistant Sublines MIA-602	MEK/ERK 1/2 PI3K/Akt	Anti-Proliferative, Apoptotic, Decreased Cell Survival, Inhibited ERK and AKT Signaling	[16,57,58,59,60]
Non-Small Cell Lung Carcinoma, Pancreatic Cancer, Gastric Cancer, Colon Cancer, Breast Cancer, Ovarian Cancer, Prostatic Cancer, Glioblastoma AVR-352, AVR-353	PAK1/STAT3, cAMP/cAMP response element-binding protein, Cyclin D_1_/D_2_, CDK4 and CDK6, and p27^kip1^	Anti-proliferative, Apoptotic, Decreased cell survival, Anti-tumorigenic, Cell cycle arrest, Inhibited PAK1/STAT3	[56]
Small and Non-Small Cell Lung Carcinomas MIA-602	cAMP/cAMP response element-binding protein, Cyclin D_1_/D_2_, CDK4 and CDK6. STAT3/PAK1 and p27^kip1^	Decreased cell survival, Apoptotic, Anti-proliferative, Cell cycle arrest, Inhibited STAT3/PAK1, Anti-tumorigenic	[34]
Mammary Cancer, Prostatic Cancer, Pancreatic Cancer MZ-5-156	GHRH-induced cAMP release VIP-induced cAMP release	Rapid and sustained cAMP- release inhibition with delayed cell responsiveness for several hours post-treatment	[39]
Retinoblastoma MIA-602	MEK/ERK 1/2 Caspase 3	Apoptotic, Anti-proliferative, Inhibited ERK signaling, Upregulated Caspase 3	[43]
Epithelial Ovarian Cancer JV-1-36 MZ-5-156	IGF-II	Anti-tumorigenic, Anti-proliferative, Decreased serum GH, Competitively inhibited GHRH-R and decreased *IGF-II* mRNA	[44]
Colon Cancer MZ-4-71	IGF-II	Apoptotic, Anti-proliferative, Decreased *IGF-II* mRNA and total IGF-II production	[45]
Pancreatic Cancer MZ-4-71 MZ-5-156	IGF-II	Anti-proliferative, Anti-tumorigenic, Reduced *IGF-I* mRNA and IGF-II production	[42]
Androgen-Independent Prostatic Cancer JMR-132	MEK/ERK 1/2 PI3K/Akt	Apoptotic, Anti-proliferative, Inhibited ERK and AKT signaling, Anti-tumorigenic	[41]
Triple-Negative Breast Cancer MIA-602	INF-γ, IL-1α, IL-4, IL-6, IL-8, IL-10, TNF-α	Reduced expression of inflammatory cytokines	[36]
Glioblastoma, Breast Carcinoma, Small Cell Lung Carcinoma, Non-Small Cell Lung Carcinoma MZ-5-156	hTRT modulation	Anti-tumorigenic, Decreased telomerase activity, Decreased *hTRT* gene expression	[46]
Non-Hodgkin’s Lymphoma MZ-5-156 MZ-J-7-138	IGF-I	Anti-tumorigenic, Anti-proliferative, Apoptotic, Decreased serum IGF-I and *IGF-1* mRNA expression, Decreased bFGF production	[51]
Glioblastoma, Estrogen-Independent Breast Cancer, Clear Cell Ovarian Cancer MIA-602	Metalloprotease-2 and metalloprotease 9, caveolin-1, E-cadherin, NF-κB, β-catenin	Reduced cell viability, Decreased cell adhesion, Lowered tumor cell invasiveness, Inhibited cell motility, Decreased metastatic potential	[37]
Renal Adenocarcinoma MZ-4-71	GH, IGF-1, IGF-II,	Anti-tumorigenic, Lowered GH, IGF-I, IGF-II levels, Anti-proliferative	[47]
Androgen-Sensitive Prostate Cancer, Androgen-Independent Prostate Cancer JV-1-38	VEGF, IGF-I/II	Anti-tumorigenic, Modulated expression of IGF-I/II and VEGF, Decreasing mRNA and production of growth factors.	[40]
Gastric Cancer MIA-602	PAK1-STAT3/NF-κB	Anti-tumorigenic, Anti-proliferative, Decreased cell viability, Lowered serum GHRH levels, Downregulated PAK1-STAT3/NF-κB axis signaling	[48]
Estrogen-Independent Breast Carcinoma JV-1-36	GH/IGF-independent mechanism, yet to be elucidated	Anti-tumorigenic, Reduced metastatic incidence, Anti-proliferative	[38]
Osteosarcoma MZ-4-71	GH, IGF-I	Anti-tumorigenic, Anti-proliferative, Reduced serum IGF-1 and serum GH	[49]
Neuroendocrine Lung Non-Small Cell Carcinoma JV-1-36	VEGF	Reduced VEGF production, Anti-proliferative	[35]
Endometrial Adenocarcinoma, Colorectal Adenocarcinoma, Prostatic Cancers, Renal Cell Carcinoma, Diffuse Mixed B Cell Lymphoma MIA-602	GH-independent growth inhibition MEK/ERK 1/2 PI3K/Akt	Anti-proliferative, Anti-tumorigenic, Inhibited ERK and AKT signaling	[50]

## 3. GHRH Receptor-Targeted Therapy in AML and APL: Pre-Clinical Efficacy of MIA-602 in Drug-Resistant Leukemia

In lieu of the prior evidence, Jimenez et al. sought to examine the presence of pGHRH-R and cancer-related SV1 variant in established AML cell lines, K-562, THP-1, KG-1, and KG-1α. KG-1 gave no evidence of having either receptor, and THP-1 showed only pGHRH-R, whilst K-562 and KG-1α showed the presence of both the pGHRH-R and SV1 isoforms [57]. All cell lines, including U937 in a later study, were incubated with increasing concentrations of MIA-602 ranging from 0.05 μmol/L to 5 μmol/L in vitro and were assessed for the impact on cell viability and proliferation, as well as effect on apoptosis at 24 and 48 h post-therapy. The results indicate that MIA-602 at 5 μmol/L produced a significant dose- and time-dependent reduction in cell proliferation, cellular viability, and induced pro-apoptotic pathways increasing overall apoptosis, KG-1 being an exception to this [57].

Additionally, U937, KG-1α, and K-562 cells were cultured to create doxorubicin-selected derivatives. Both non- and doxorubicin-resistant clones were treated in vitro with MIA-602 alone or combined with doxorubicin at varying concentrations ranging from 0.05 to 5 μmol/L and 0.005 to 0.05 μg/mL respectively [16]. Of note, combination therapy showed significant synergy, with potent anti-neoplastic effects realized at the maximal concentration of 5 μmol/L and 0.05 μg/mL respectively. Doxorubicin-resistant clones subjected to MIA-602 monotherapy still showed significant time- and dose-dependent cancer-inhibition, representing a novel therapeutic modality functioning independent of traditional chemotherapeutic mechanisms [16].

Xenografted tumors by s.c injection of the KG-1α, K-562, and THP-1 lines in athymic nude mice were all confirmed to still express either pGHRH-R or SV1 receptors by both Western blot analysis and immunohistochemistry staining. MIA-602 was then administered s.c. at a dose of 10 μg twice per day, for a month, yielding significant tumor volume reductions and a prolonging of tumoral doubling times [57]. Subsequent work utilized doxorubicin-resistant K-562 cells xenografted into athymic nude mice to model chemotherapy-resistance in tumors in vivo and were subject to treatment by s.c. injection with 10 μg of MIA-602 twice per day, for a month. MIA-602 therapy resulted in a significant reduction in average tumor volume compared to the control group. Upon examination of gross anatomy, no visible changes due to cytotoxic effects were seen in treated animals, and the native organs and body mass presented the same as untreated, non-tumor-bearing mice [16].

These findings in AML models prompted investigation into whether GHRH antagonism could similarly overcome resistance mechanisms in APL, particularly in the context of ATRA/ATO resistance (RAA) [59]. The promyelocytic leukemia cell line NB4 was chosen to model APL and had the presence of GHRH-R verified by Western blot analysis in the parental cell line, and ATRA/ATO-resistant subline. The effect of MIA-602 treatment in vitro on cellular proliferation, cell survival, and apoptosis was measured with increasing concentrations of the antagonist ranging from 0.05 to 5 μmol/L at the 24 and 48 h mark. NB4 and NB4-RAA showed the highest anti-neoplastic effects at the maximal dose, 48 h post-therapy. MIA-602 treatment caused a significant dose- and time-dependent reduction in both cellular survival and cell proliferation rates, whilst vastly increasing the number of apoptotic cells [59]. Following this both NB4 and NB4-RAA cells were xenografted into athymic nude mice by s.c. injection to study MIA-602’s effects on APL and ATRA/ATO-resistant APL in vivo. The tumor-bearing mice were given MIA-602 at a dose of 10 μg twice per day, for 30 days. This resulted in significant average tumor volume reduction in both NB4 and NB4-RAA tumors after MIA-602 therapy. There were no visible sequelae of any cytotoxic effects on peripheral tissues upon macroscopic examination, and there were also no significant differences between the weight of organs, or of the bodyweight of treated mice and non-tumor-bearing mice [59].

Recently, a more clinically aligned acetate salt form of MIA-602, MIA-602 (Ac), was formulated in order to circumvent unexpected issues associated with the synthetic methodology of this peptide that prevented subsequent translation into human studies. The chemical process required for cleaving this peptide from its synthesis resin uses a solvent containing TFA during elution. This introduces residual TFA to the peptide formulation and was deemed not acceptable for human studies given potential subcutaneous toxicity from residual TFA exposure. To ameliorate this, the peptides can be passed through a carbonate ion-exchange resin column, and dilute acetic acid is added to the resulting elute prior to lyophilization. This yields MIA-602 (Ac) and removes the previous concerns for toxicity related to TFA burden [61,62]. MIA-602 (Ac) was tested against hormone-sensitive prostate cancer, castration-resistant prostate cancer, and neuroendocrine prostate cancer models in vitro and retained biological activity and signaling effects comparable to its TFA formulation suggesting preserved pharmacodynamics and therapeutic potential [61].

Similarly to previous investigative work evaluating MIA-602 against AML models, MIA-602 (Ac) was tested against K-562 and its doxorubicin-resistant analog, as well as NB4 and NB4-RAA such that retention of previous anti-leukemic efficacy could be measured. K562 and its anthracycline-resistant analog, as well as NB4 and NB4-RAA were cultured with increasing doses of MIA-602 (Ac) ranging from 0.05 to 5 µmol/L, measuring cell viability at both 24 h and 48 h post-therapy, as done in previous experiments. As seen with MIA-602 in the past, MIA-602 (Ac) monotherapy maintained a significant dose- and time-dependent anti-leukemic effect across both non- and drug-resistant AML/APL analogs in vitro [63]. Of note, the evidence for MIA-602 (Ac) as a therapeutic modality in AML is entirely pre-clinical.

## 4. Implications for Drug-Resistance in AML

Our current understanding of leukemogenesis is that it occurs as a series of genetic mutations that are acquired and propagate across hematopoietic stem cells and progenitor cells, which afford these now leukemic cells an irregular and pathological capacity to self-renew and generate neoplastic clones. These leukemogenic events include but are not limited to modifications in tumor suppressor genes, RNA splicing, cytokine signaling pathways, epigenetic regulators, transcription factors and their cognate receptors, chromosomal structures, metabolic enzymes, and nucleoplasmin shuttle proteins as is the case for AML [1]. It is also known that identification of recurrent mutations and genomic abnormalities can impact prognostic assessment and carry crucial therapeutic value, making it standard practice during the diagnosis and classification of AML [13,64]. However, due to the heterogenous nature and the phenotypic continuum of presenting leukemias, this might still come with incongruencies between leukemic pathobiology and molecular signatures. Typically, screening for genetic aberrations, as recommended by the European LeukemiaNet (ELN) and National Comprehensive Cancer Network (NCCN), seeks to rapidly define actionable lesions like *FLT3* and *NPM1* mutations and is supplemented by broader myeloid next-generation sequencing panels that capture genes such as *IDH1/2*, *TP53*, *RUNX1*, and *CEBPA* serving to further guide AML classification, risk-stratification, and therapeutic management [11]. Notably, the 2022 5th edition W.H.O. classification on myeloid neoplasms references the prevalence of *ASXL1*, *DNMT3A*, and *TET2* mutations in over half of patients with myeloproliferative neoplasms, which can subsequently develop into AML [3]. These molecular markers are consistent with pre-malignant clonal hematopoiesis and can be used as information to further monitor the disease through next-generation sequencing techniques, although their role in AML is still under investigation and of much debate [3]. Of the previously mentioned lesions, *FLT3* mutations have emerged as a critical focal point for both risk-stratification and targetable intervention.

The *FLT3* gene encodes a type III receptor tyrosine kinase and bears similar homology to other receptors of its same class, namely KIT, platelet-derived growth factor receptor, and colony-stimulating factor 1 receptor. Although its ligand (FLT3-L) has been identified in several tissues, the receptor is more localized and is highly expressed on the surface of both hematopoietic stem cells, and progenitor cells [65]. *FLT3* has been demonstrated to be essential for the proper functioning of hematopoiesis, governing the maturation and production of blood cells, unsurprisingly being one of the most common mutations seen in AML. The incidence rate of *FLT3* mutations sits at ~ 30% of AML overall. The most prevalent *FLT3* aberration is the in-frame internal tandem duplication (*FLT3-ITD*) presenting in approximately 20–30% of AML overall, or as a point mutation in the tyrosine kinase domain of *FLT3* (*FLT3-TKD*) occurring in about 5–10% of AML cases overall [66,67,68]. Such alterations lead constitutive activation of the receptor, uncontrolled proliferation, and enhanced survival of leukemic cells, with *FLT3-ITD* variants usually meaning a poorer patient prognosis and a higher incidence of relapse [69]. The prognostic influence of *FLT3-TKD* mutations still remain to be fully elucidated and are not considered in risk assessments set forth by the ELN [11,68]. *FLT3* mutations are found at the time of AML diagnosis or can even arise/disappear once at relapse. Additionally, *FLT3* are heterogenous mutations varying from patient to patient due to differences in point mutations for *FLT3-TKD* or by the allelic burden and varied duplication lengths found in *FLT3-ITD* variants obviating potential differences in patient outcomes [70]. Despite patient variability and its potential to skew the efficacy of FDA-approved FLT3-inhibitors, targeting of FLT3 receptors has led to significant clinical breakthroughs in the treatment of AML [70]. The result of this, in the most recent ELN 2022 guidelines, has been a re-classification of *FLT3-ITD* mutations without adverse-risk genetic lesions from the adverse-risk category to that of intermediate risk, irrespective of allelic ratio and/or the presence of an *NPM1* co-mutation, reflecting the effectiveness of FLT3 inhibitors and the improvements seen in overall survival (OS) and relapse rates of patients [11]. FLT3 signaling is known to stimulate the PI3K/AKT/mTOR and RAS/MAPK/ERK1/2 and JAK/STAT5 pathways [71,72,73]. Once mutated, the constitutively activated receptor permits unregulated growth signaling to drive leukemogenesis by suppressing apoptotic signals, enhancing cellular growth and proliferation, whilst simultaneously skewing differentiation programs halting cells in progenitor-like states, greatly expanding leukemic blasts throughout the peripheral blood and bone marrow [66].

The clinical efficacy of FLT3 inhibition as a viable therapeutic target has been validated by its widespread success, and several FDA-approved pharmaceutical agents including Midostaurin-based therapy in newly diagnosed *FLT3*-mutated AML [73,74,75]. Gilteritinib is indicated as a monotherapy for R/R *FLT3-ITD* or *FLT3-TKD* mutated AML [76]. Gilteritinib, a type I inhibitor like Midostaurin, is capable of inhibiting both active and inactive conformations of FLT3 yet differs significantly from previous generation inhibitors in that it is not as broad a multi-kinase inhibitor and is therefore a “cleaner” therapeutic agent. Gilteritinib can act against a wide breadth of *FLT3* mutations and conformational states with superior selectivity conferring it partial capacity to circumvent intrinsic FLT3 inhibitor resistance [74]. The newest FDA-approved FLT3 inhibitor is an even more selective second-generation type II inhibitor, only capable of binding to FLT3 in its inactive confirmation thus exhibiting the highest efficacy against *FLT3-ITD* AML whilst failing often to inhibit *FLT3-TKD* mutations, known as Quizartinib. As such, Quizartinib-based approaches are notoriously vulnerable to therapeutic resistance via *FLT3-TKD* mutations [68,73]. Quizartinib is FDA-approved for newly diagnosed *FLT3-ITD* AML during induction, consolidation, and maintenance regimens [77]. Despite advancements made in FLT3 inhibitor-based approaches with drugs like Gilteritinib and Quizartinib, resistance to FLT3 inhibitors, especially in a refractory setting, remains of upmost concern. Currently responses seen in patients are often transient and ultimately lead to relapse [68,73].

Resistance to FLT3 inhibitors can occur via intrinsic mechanisms such as secondary *FLT3* mutations arising *de novo*, resulting in an altered FLT3 receptor shape or diminished kinase activity, in turn worsening the drug binding kinetics or intended inhibition of signaling by the administered drugs. Alternatively, inherent sub-clonal expansion of leukemic blasts capable of sustaining off-target pathway signaling resulting in parallel or downstream activation of PI3K/AKT/mTOR, RAS/MAPK/ERK1/2, or JAK/STAT5 signaling independent of FLT3 receptor involvement can serve as a route for intrinsic resistance [70,73,74]. In contrast, resistance can be extrinsic, as exemplified by the bone marrow microenvironment. In response to chemotherapy, mesenchymal stromal cells may produce compensatory surges of FLT3-ligand, outcompeting FLT3 inhibitors, and thus permitting pro-survival signaling in leukemic blasts and providing a way for leukemic cells to survive. On the other hand, FLT3 inhibitor efficacy can be extrinsically lowered through secretion of other growth factors by marrow stromal cells in response to chemotherapy, which can be capable of stimulating the PI3K/AKT, MAPK/ERK or JAK/STAT pathways and effectively overcoming suppressive signaling [66,73].

To our knowledge, the current literature does not present any direct evidence showing MIA-602 or GHRH antagonists with capability to modulate FLT3 receptor phosphorylation, *FLT3-ITD/FLT3-TKD* signaling, or reverse FLT3-inhibitor resistance specifically. The rationale for discussing *FLT3*-mutated AML is not that MIA-602 directly targets FLT3, but instead to highlight the convergence of several downstream or parallel survival pathways modulated by GHRH antagonism, such as PI3K/AKT, RAS/RAF/MEK/ERK, STAT-family signaling, inflammatory cytokine signaling, and apoptosis escape which are also pathways implicated in FLT3-inhibitor resistance [57,59]. GHRH antagonists when viewed in this light could plausibly serve as a potential resistance modifying strategy to lower or alter an *FLT3*-mutated AML’s capacity to resist therapeutic intervention. Yet, the evidence while hypothetical and needing further experimental validation, could prove useful given the demand for novel therapeutic modalities and pharmaceutical answers to drug-resistant disease. Continuous efforts in the development of GHRH antagonists have yielded increasingly potent anti-neoplastic agents with highly favorable toxicity profiles, increasing their potential for future implementation as a novel therapeutic modality [50].

Unlike FLT3 inhibitors, GHRH antagonists have yet to be explored in the context of clinical trials, although preliminary data has found bioactive GHRH-R in nine out of nine primary samples from AML patients, supporting further investigation for translational feasibility from animal to human investigations [57]. The AML of each patient was classified as follows: 1—AML with t(9;11)(p22;q23) *MLLT3::KMT2A* and t(4;15)(p31;q22) *TMEM154::RASGRF1*; 2—AML with *IDH1* (132R/C) and *DNMT3A* mutations; 3—AML minimally differentiated with trisomy 13; 4—AML with *NRAS* and *TET2* mutations; 5—AML with *NPM1* mutation; 6—AML with *FLT3*-*ITD*; 7—AML with *NRAS* mutation; 8—AML with *IDH2* (140R/Q) mutation; and 9—AML with *CSF3R* and *TET2* mutations [57]. The detection of GHRH-R in all nine samples does offer support for further investigation of GHRH-R receptor expression across more clinical AML subtypes; however, due to the limited sample size and heterogenous molecular profiles, the data does not reveal much as to the role GHRH antagonism would play at large, nor does it establish anti-leukemic activity specific to *FLT3*-mutated AML. Future studies should compare *FLT3-ITD*, *FLT3-TKD*, and *FLT3*-wild-type AML clinical samples versus established *FLT3*-mutated cell lines such as MOLM-13 and MV4-11 alongside *FLT3*-wild-type controls all stratified by GHRH-R/SV1 expression. In addition, they should compare functional readouts of phosphorylated signaling mediators such as p-FLT3, p-STAT5, p-AKT, and p-ERK, release of leukemogenic cytokines, stromal factors, or expression of proteins related to apoptosis. This experimental set-up could help facilitate understanding resistance in *FLT3*-mutated AML biology, might apply to resistance more broadly, and could even demonstrate pathway modulation due to overlapping signaling networks.

## 5. Limitations and Translational Barriers

Although various in vitro and in vivo models of AML, including APL, in addition to clinical samples, have indicated the presence of GHRH-R and revealed MIA-602 to produce a significant time- and dose-dependent reduction in cellular proliferation, cell survival, and xenograft tumoral growth, the evidence is inherently limited. The evidence base is largely reliant on data derived from established leukemia cell lines, their drug-selected resistant sublines, and subcutaneous xenograft models in immunodeficient mice, thus constraining the models and likely not fully capturing AML clonal heterogeneity, immune interactions, marrow niche biology, or pharmacological complexities associated with treatment administration and efficacy. The data recorded thus far should serve as an initial proof of principle of GHRH-R anti-cancer activity under these designed experimental parameters and are not generalizable to broader AML biology, *FLT3*-mutated AML modulatory capacity, or ability of GHRH antagonists to address therapeutic resistance across AML subtypes [57,59,78].

Another relevant limitation to this work is the current incomplete characterization of GHRH-R and SV1 expression across defined clinically defined AML subsets. The prevalence of GHRH-R/SV1 expression, the clinical significance once targeted, and correlational biological data from large, reputable sources are ill-defined, and as of now, largely speculative until reproducibly measured and widely reported throughout the literature. The only available clinical data was encouraging but limited by sample size and heterogenous leukemic biology. Future studies should characterize GHRH-R and SV1 expression across larger AML cohorts stratified by clinically relevant genetic features, including *FLT3-ITD*, *FLT3-TKD*, *FLT3*-wild-type, *NPM1*, *IDH1/2*, *TP53*, RAS-pathway alterations, *KMT2A* rearrangements, and R/R disease state. Receptor detection only will not suffice, genetic knockdown studies, ligand-competition studies, rescue experiments, and direct pharmacodynamic read outs are also necessary in order to more accurately assess GHRH antagonism’s functional relevance in AML.

**Figure 1:**
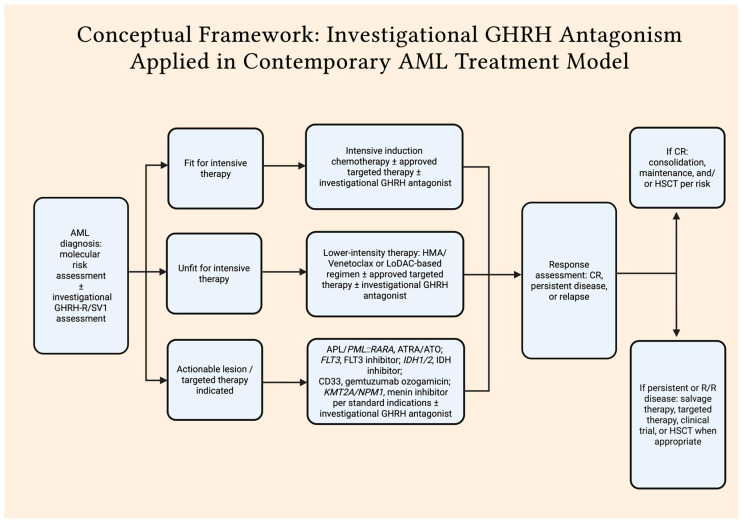
Conceptual framework of a contemporary AML treatment model with the inclusion of GHRH antagonist-based therapy. GHRH-R and SV1, denoting splice variant 1, the principal tumor-associated GHRH-R variant implicated in GHRH bioactivity, would be assessed during AML diagnosis work-up. Use of GHRH antagonist would later be considered during standard-of-care AML therapy regimens. GHRH antagonists shown here are currently at the pre-clinical stage and not established AML therapy. AML, acute myeloid leukemia; GHRH-R, growth hormone-releasing hormone receptor; SV1, splice variant 1; CR, complete remission; R/R, relapsed or refractory; HSCT, hematopoietic stem cell transplantation; HMA, hypomethylating agent; LoDAC, low-dose cytarabine; APL, acute promyelocytic leukemia; *PML*, promyelocytic leukemia; ATRA, all-trans retinoic acid; ATO, arsenic trioxide; *FLT3*, fms-like tyrosine kinase 3; *IDH1/2*, isocitrate dehydrogenase 1/2; *KMT2A*, lysine methyltransferase 2A; *NPM1*, nucleophosmin 1. Created in BioRender. Costoya, J. (2026), https://BioRender.com/c44h727 (accessed on 15 June 2026).

Lastly, although animal proof-of-concept studies might not have shown overt treatment-associated toxicity, human pharmacological and safety data for MIA-602 is currently unavailable. Future translational studies should look to prioritize patient-derived xenograft AML models, normal CD34-positive hematopoietic toxicity assays, and molecularly stratified testing across *FLT3*-mutated versus *FLT3*-wild-type models. Direct biochemical and functional readouts including p-FLT3, p-STAT5, p-AKT, p-ERK, apoptotic markers, stromal-rescue assays, and receptor-dependence studies will be needed to determine whether GHRH-R/SV1 antagonism modifies resistance-associated signaling. Figure 1 provides a conceptual framework for the potential investigational GHRH-R/SV1 assessment and treatment decision point inclusions within a contemporary AML treatment model.

## 6. Conclusions

As it stands, evidence regarding the application of GHRH antagonists, such as MIA-602, to AML remains pre-clinical and supports continued investigation. MIA-602 has demonstrated anti-leukemic activity against in vitro AML and APL models, as well as animal xenograft models in vivo, and their drug-resistant sublines. However, the therapeutic potential of GHRH antagonists, as they relate to broader AML subtypes is not yet fully understood, and key translational parameters such as pharmacokinetic studies, dose optimization, safety, and clinical efficacy remain to be established.

Biomolecular pathways implicated in FLT3 inhibitor resistance can involve PI3K/AKT, MAPK/ERK, and JAK/STAT signaling, as well as inflammatory cytokine modulation, microenvironmental stromal interactions, and aberrant apoptotic signaling networks. Several of these pathways share similar signaling networks to those reported by studies on MIA-602 and other GHRH antagonists. To date, no GHRH antagonist has been evaluated in *FLT3*-stratified AML models. No published evidence directly demonstrates GHRH antagonists possessing the ability to modulate FLT3 activation, *FLT3-ITD/TKD* signaling, or resistance to FLT3 inhibitors. Accordingly, GHRH antagonists should not be framed as an FLT3-targeted therapy. The potential relevance of MIA-602 to *FLT3*-mutated AML remains indirect and hypothesis generating. GHRH antagonists might serve as a resistance modifying strategy, due to shared overlap and potential modulation of signaling networks implicated in FLT3 inhibitor resistance.

Due to the absence of correlative clinical data, formal toxicology reports, and larger studies defining how GHRH signaling presents across larger *FLT3*-mutated AML cohorts, its clinical relevance remains uncertain. Future studies on GHRH antagonism should define GHRH-R/SV1 expression across molecularly annotated AML subtypes, and test MIA-602 against both *FLT3*-mutated and *FLT3*-wild-type AML models. Comparing direct biochemical and functional readouts between these groups across signaling networks implicated in FLT3 inhibitor resistance could clarify whether GHRH-R/SV1 antagonism has relevance for future AML resistance-aimed research. In the end, until more is known regarding AML-specific clinical efficacy, human pharmacokinetics, optimal dosing schedules, and drug safety data is formalized, GHRH antagonists like MIA-602 should be regarded as a hypothesis-generating pre-clinical research direction, rather than an established therapeutic modality for AML.

## Data Availability

No new data were created or analyzed in this study. Data sharing is not applicable to this article.

## Abbreviations

The following abbreviations are used in this manuscript:

GHRH	Growth Hormone-Releasing Hormone
AML	Acute Myeloid Leukemia
GHRH-R	Growth Hormone-Releasing Hormone Receptor
APL	Acute Promyelocytic Leukemia
ATRA	All-*Trans*-Retinoic Acid
ATO	Arsenic Trioxide
IGF-I	Insulin-Like Growth Factor 1
SV	Splice Variant
pGHRH-R	Pituitary Growth Hormone-Releasing Hormone
RAA	ATRA/ATO Resistance
ELN	European LeukemiaNet
NCCN	National Comprehensive Cancer Network
*FLT3*	Fms-Like Tyrosine Kinase 3
FLT3-L	Fms-Like Tyrosine Kinase 3-Ligand
*FLT3-ITD*	Fms-Like Tyrosine Kinase 3 In-Frame Internal Tandem Duplication
*FLT3-TKD*	Fms-Like Tyrosine Kinase 3 Tyrosine Kinase Domain
R/R	Relapsed or Refractory
OS	Overall Survival
TFA	Trifluoroacetic Acid
MIA-602 (Ac)	Acetate Salt Form of MIA-602
HMA	Hypomethylating Agent
LoDAC	Low-Dose Cytarabine
*PML*	Promyelocytic Leukemia
HSCT	Hematopoietic Stem Cell Transplant
PKA	Protein Kinase A
GPCR	G Protein-Coupled Receptor
AKT	Protein Kinase B
PI3K	Phosphoinositide 3-Kinase
cAMP	Cyclic Adenosine Monophosphate
*IDH 1/2*	Isocitrate Dehydrogenase 1/2
*CBF*	Core Binding Factor
*NPM1*	Nucleophosmin 1
*KMT2A*	Lysine Methyltransferase 2A
